# Understanding mother-to-child transmission of HIV among mothers engaged in HIV care in Kenya: a case report

**DOI:** 10.1186/s13006-024-00622-3

**Published:** 2024-02-24

**Authors:** Emily L. Tuthill, Belinda C. Odhiambo, Ann E. Maltby

**Affiliations:** 1 Department of Community Health Systems, School of Nursing, University of California, 12 Hayfield Rd, Etna, San Francisco, CA NH 03750 USA; 2 Global Programs for Research and Training, University of California, San Francisco, CA USA

**Keywords:** Case report, Exclusive breastfeeding, Global AIDS strategy, HIV, Kenya, Mother to child transmission of HIV

## Abstract

**Background:**

Mother-to-child transmission of HIV, which may occur in utero, during birth, or through breastmilk, is now largely preventable with the advancement of HIV testing and treatment for women and their infants. Globally, great progress has been recorded over the years, with a 58% decline in new infections in children from 2010 to 2022. Currently, Kenya is among the countries with the highest rates of mother-to-child transmission of HIV despite consistent efforts to promote prevention of mother to child transmission strategies.

**Methods:**

This case report presents the experiences of a woman, engaged in HIV care in Kenya, whose baby contracted HIV. The data used to describe this case come from surveys, provider notes, health records, observational notes, notes from phone call consultations, and one in-depth interview. All data sources were carefully reviewed, compared and complied to describe the timeline of events and context of the participant’s experience.

**Results:**

We found multiple factors which may have contributed to this case of mother-to-child transmission of HIV. Antenatal care was initiated late in pregnancy (during the third trimester), and as a result, HIV diagnosis and treatment also occurred late in pregnancy. In addition, a lack of coordination between the clinic providing antenatal care and HIV treatment, and the hospital providing labor and delivery services led to breastfeeding initiation prior to the administration of infant HIV prophylaxis medications. Finally, poor maternal adherence to HIV medications went undetected and unaddressed until it was revealed by routine viral load monitoring three months after initiating HIV treatment (more than two months postpartum).

**Conclusions:**

Our case report shows the continued need for more intensive and integrated care for mothers living with HIV and their infants including support for pregnant women newly diagnosed with HIV, coordination of perinatal and HIV care, provisions for routine monitoring of HIV medication adherence, intensive follow-up care including point of care testing for HIV exposed infants and in person breastfeeding support. Our case report contributes an important perspective especially in light of the current UNAIDS Global AIDS Strategy which recently inspired the Global Alliance to end AIDS in Children.

## Background

In 2022, there were around 1.2 million pregnant women and girls living with HIV [1]. Without any treatment, 15 to 30% of HIV exposed infants become infected during pregnancy, labor, or delivery with an additional 5–15% contracting HIV during breastfeeding [2]. However, when mothers’ viral loads are suppressed by medications during the breastfeeding period, the rate of mother to child transmission of HIV (MTCT) may be less than 2% [3]. Since 2011, international efforts have made great progress towards reducing MTCT through increased access to preventative services and antiretroviral therapy (ART) [4]. As a result, there has been a 58% decline in new infections in children from 2010 to 2022 [5]. Yet, in 2022, there were 130,000 new HIV infections among children, and global progress towards the elimination of MTCT has stagnated as the proportion of pregnant and breastfeeding women living with HIV who receive ART has remained at around 80% since 2015 [6]. In addition, after only slowly declining over several years, the global rate of MTCT was 11% in 2021 and 2022 [7]. UNAIDS’s Global AIDS Strategy 2021–2026 called attention to stalled progress towards ending AIDS in Children inspiring key stakeholders, including the World Health Organization, to propose a Global Alliance to end AIDS in Children through informed, renewed efforts [4, 8]. In February of 2023, 12 African countries, motivated by the Alliance, created and signed the Dar es Salaam Declaration committing to a set of actions to end AIDS in Children [9].

Kenya, a country where 890,000 women are living with HIV, was one of the countries that signed the Dar es Salaam Declaration thereby committing to provide treatment and support for sustained engagement in care to all pregnant and breastfeeding women [9, 10]. Thanks to previous efforts by the Kenyan Ministry of Health and partner agencies, 91% of pregnant women living with HIV (WLWH) were already receiving ART in 2021 [10, 11]. Yet, the rate of MTCT in Kenya remains unacceptably high at 8.9% with an estimated 5,200 new HIV infections among children (0–14) in 2021 [10]. To eliminate MTCT in Kenya, a detailed understanding of the multi-level factors contributing to MTCT in this setting is necessary, as WLWH encounter specific challenges to preventing MTCT including late initiation of antenatal care leading to delayed HIV screening, struggles with maternal adherence to ART, difficulties remaining engaged in care, and barriers to adhering to infant feeding recommendations [12–16]. To understand how MTCT of HIV still occurs in this setting, we present the case of a mother living with HIV whose baby tested positive for HIV at four weeks postpartum.

## Methods

Information about this case was collected from 23 February 2022 to 23 August 2022 during the feasibility trial of an intervention aimed at addressing food insecurity and supporting optimal infant feeding. The trial was conducted at a sub-County Hospital in western Kenya where prevention of mother to child transmission of HIV (PMTCT) services were provided including ART and viral load testing for women, HIV testing and prophylaxis medications for infants and counseling and education related to living with HIV and the care of HIV exposed infants. Our research team who planned and carried out the feasibility trial consisted of the Primary Investigator based in the United States (ET), a Research Coordinator (BO), a Research Assistant and a Lactation Specialist based in Kenya, and a Research Coordinator based in Denmark (AM). Participants in the intervention received personalized breastfeeding support from the Lactation Specialist until three months postpartum. Data compiled to form this case report include clinical notes from the Lactation Specialist, observation notes from the supportive sessions, phone call consultations documented by the on-site Research Coordinator, questionnaire data collected through electronic surveys, communications with healthcare providers, health records and an in-depth exit interview with the participant at approximately six months postpartum.

## Case presentation

Susan (a pseudonym) was a shy, 32-year-old, unemployed, mother of two with a primary school education. She lived in informal housing in an urban area with her husband and was having a planned pregnancy. We met Susan at her 2nd antenatal care visit where she reported she was 29 weeks pregnant (estimated based on her last menstrual period). Susan had been diagnosed with HIV and started on ART at her first antenatal visit, approximately six weeks prior to our meeting. She reported that she had disclosed her new diagnosis to her husband who she believed was HIV negative. When surveyed about her adherence to ART during this initial visit, Susan reported perfect adherence. Since the first viral load test for people newly diagnosed with HIV in this setting is done three months after the initiation of ART, Susan was yet to have her viral load measured [17]. Susan was planning to exclusively breastfeed her baby for the first six months postpartum as recommended, but was concerned she may not produce enough milk [17, 18].

**Fig. 1:**
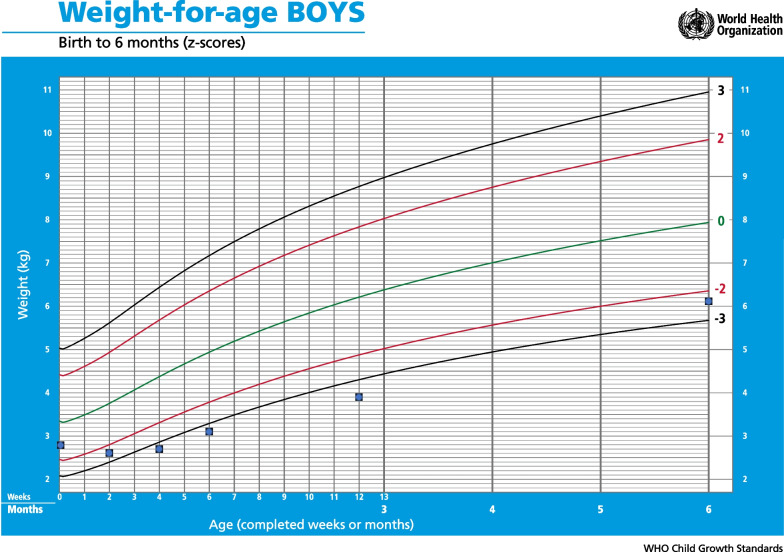
The birth weight was collected from Susan’s infant’s medical record, the other infant weights were measured on a digital scale by our Research Coordinator at each postpartum research encounter. The weight-for-age z-scores plotted on this chart were then calculated using World Health Organization child growth standards [19]

About a week after we met Susan, she gave birth. This was just over seven weeks after being diagnosed with HIV, and after having attended only two prenatal care visits. Susan gave birth nearly two months earlier than expected. Her son weighed 2.8 kg (weight for age z-score = -1.18; see Fig. 1 for weight-for-age z-scores at each timepoint as compared to WHO standards), but showed no other signs of prematurity, indicating a possible miscalculation of her due date.

Susan delivered a baby boy via normal vaginal delivery at a hospital 20 min (via motorcycle) from her home where skilled providers were present. This was a different facility from the PMTCT clinic where she had been receiving antenatal and HIV care. At the time of delivery, Susan did not have HIV prophylaxis medications for her baby (these are usually given to women at the PMTCT clinic about a month before their due date), nor did hospital staff provide Susan’s newborn with HIV prophylaxis medications. In fact, it was unclear (based on Susan’s description) whether or not the providers who cared for her during labor, delivery and immediately postpartum knew she had been recently diagnosed with HIV. Susan reported she and her baby experienced no complications. She initiated breastfeeding in the hospital, and was discharged home with her son after one day.

Four days after delivery, Susan met with the Lactation Specialist who noticed immediately that she was having challenges with breastfeeding (see Table 1 for an overview of the lactation support provided). Breastfeeding challenges aside, it was very concerning at this time to discover that Susan had not yet received her baby’s HIV prophylaxis. After meeting with the Lactation Specialist, Susan attended her regularly scheduled appointment at the PMTCT clinic where she ultimately received HIV prophylaxis medication for her baby.

**Table 1:** Research encounters and lactation support provided

**Pregnancy**
Susan met with the Research Coordinator for the first time. She consented to participate in the intervention feasibility trial, and survey data were collected including demographics and information about Susan’s health and adherence to ART.
**4 days postpartum**
Susan met with the Lactation Specialist for the first time. She reported she had been breastfeeding exclusively, but complained of red, sore nipples, and that her baby was breastfeeding “too much” which made her feel she was not producing enough milk. The Lactation Specialist assessed that Susan had difficulty positioning her baby and achieving an effective latch. She helped Susan to find a more comfortable position and deeper latch thereby reducing nipple pain and improving milk transfer. She also reassured Susan about her milk supply by hand expressing milk from her breast and provided information about the importance of feeding on demand to stimulate milk production.
**2 weeks postpartum**
Susan expressed continued concern that she was not producing enough milk. The Lactation Specialist assessed that Susan was still struggling with positioning and latch, she worked with Susan to improve her breastfeeding technique and provided reassurance. The Lactation Specialist was concerned about postpartum depression as Susan seemed detached from her baby and was not maintaining eye contact during breastfeeding
**4 weeks postpartum**
Susan reported that her son was frequently feeding but only for short periods. The Lactation Specialist continued to work with Susan on improving her breastfeeding technique. She also emphasized the importance of ensuring the baby receives more hindmilk (with frequent short feedings he may have been primarily taking in foremilk). Susan was receptive to the support provided at this time.
**6 weeks postpartum**
Susan mentioned worrying about her baby contracting HIV through breastmilk, and was unsure about the benefits of exclusive breastfeeding. The Lactation Specialist noticed notable improvement in breastfeeding technique especially achieving a consistently deeper and more effective latch. She continued to coach Susan to find a more comfortable position, reviewed the many benefits of breastfeeding, and reinforced the importance of maternal and infant adherence.
**3 months postpartum**
At the last supportive session, the Lactation Specialist noted Susan had a rash on her scalp and breasts and was concerned that Susan was still struggling to accept her status and/or her baby’s new diagnosis. She offered support and encouragement for continued breastfeeding as well as emphasized the importance of adherence to ART for Susan and her baby. The Lactation Specialist also provided information and support for continued exclusive breastfeeding as well as optimal infant feeding beyond six months postpartum (i.e., complementary feeding).
**6 months postpartum**
This was the final research encounter where Susan met with our Research Coordinator and participated in an in-depth exit interview.

At two weeks postpartum, Susan met with the Lactation Specialist again (see Table 1). Susan’s baby was looking sick, weak and weighing 200 g less than his birth weight (see Fig. 1). The Lactation Specialist was worried that the baby could have an infection, and the routine follow up appointment for Susan’s baby was not until six weeks postpartum. Thus, the Lactation Specialist arranged for Susan to be seen by the PMTCT clinicians immediately. After visiting the PMTCT clinic that same day, Susan reported she had been given Septrin (an antibiotic combination of trimethoprim and sulfamethoxazole) and Piriton (chlorphenamine, an antihistamine) to administer to her baby.

At four weeks postpartum, Susan’s baby still appeared unwell and thin, and now had oral thrush. He was not breastfeeding well, and his weight was still 100 g below his birth weight (see Fig. 1). Concerned about the health of Susan’s baby, the Lactation Specialist reached out to the PMTCT clinic again, this time to facilitate an early PCR HIV test for Susan’s baby (routine testing for HIV exposed infants would otherwise not occur until six weeks postpartum in this setting) [17]. Susan’s blood sample was also collected for viral load testing at this time as it had now been nearly three months since she initiated ART.

At six weeks postpartum, the Lactation Specialist assessed that Susan’s baby was looking and feeding better than in the previous visits with a weight gain of 400 g over the previous two weeks (see Fig. 1). At this time, Susan had not yet received the results of her viral load test or her baby’s HIV test. When surveyed about her adherence to ART, Susan reported not taking ART for an entire week of the preceding month when she travelled to her rural home and left her medications behind.

After Susan’s visit at six weeks postpartum, we were unable to get in touch with her by phone until nine weeks postpartum. At that time, we asked Susan to report to the PMTCT clinic team to receive newly available test results — Susan’s viral load was high at 1.7 million copies, and her baby’s PCR test was positive for HIV. The PMTCT clinic providers then met with Susan to counsel her and initiate HIV treatment (ART) for her son. The clinic providers suspected that Susan’s viral load was high due to poor adherence. They feared she had not disclosed her new diagnosis of HIV to her partner and had therefore not been taking her medications as directed. To address this, the clinic staff invited Susan to bring her partner to be tested at the clinic where they would provide counseling and support for disclosure and adherence. Susan’s partner eventually tested HIV negative, and expressed willingness to support Susan with her and their son’s adherence. Subsequently, Susan admitted to the clinic staff that she had been throwing away her ART ever since she had tested HIV positive during pregnancy.

At three months postpartum, Susan had her final visit with the Lactation Specialist. Her baby was now 1.07 kg more than his birth weight (see Fig. 1), and was looking well besides having persistent oral thrush. Susan reported she was now taking her HIV medication well.

During follow-up calls made to check on Susan and her baby at four months and five months postpartum, Susan reported that she and her baby were doing well. When she came back for her six-month appointment and exit interview, Susan and her baby looked well, she was in a good mood, laughing at times and her baby had grown considerably, now weighing 6.1 kg (see Fig. 1). Susan reported she was grateful for the sessions with the Lactation Specialist, which according to her, had really helped her bond with her baby, improve her milk supply, and remain engaged in HIV care. Referring to the supportive sessions she noted, “I was able to care for my baby according to the information I was given, and my baby improved. He is not the way he was, I fulfilled what I was taught here.” Susan’s routine viral load test measured at around this same time had markedly improved, though was still detectable at 102 copies/ml. She reported no ongoing challenges with adherence.

## Discussion

This case report outlines the circumstances surrounding one instance of MTCT which occurred in Kenya in 2022. Susan’s case provides an in-depth reporting of how MTCT is still happening, and what is needed to optimize care for WLWH and their HIV exposed infants. Late engagement in antenatal/HIV care, difficulty accepting and disclosing a new diagnosis of HIV, unrecognized non-adherence, fragmented care (e.g., giving birth at a separate facility than PMTCT clinic), and delays in HIV and viral load testing may have contributed to this case of MTCT. In contrast, sustained engagement in care, and intensive postpartum monitoring and support provided by a professional Lactation Specialist, PMTCT clinic providers, and eventually Susan’s partner may have been key to optimizing health and wellbeing for Susan and her son despite MTCT having occurred.

In 2022, more than half of new HIV infections among children occurred during pregnancy when mothers did not receive ART, stopped taking ART, or were newly infected with HIV [7]. Given Susan was more than halfway through her pregnancy when she presented for antenatal care and discovered she was living with HIV, it is possible that HIV transmission occurred during pregnancy. Women in sub-Saharan and East African countries commonly delay engagement in antenatal care which has been related to varied factors including lower maternal education, uncertainties about pregnancy status, low household income and increased birth order [20, 21]. In Kenya, approximately 82% of women deliver their babies in a health facility and 89% are attended to by a skilled provider, yet, only around 66% of women attend the recommended four or more antenatal care visits [22]. These statistics reflect Susan’s experience as this was her third pregnancy, and despite being a planned pregnancy, timely prenatal care was missing from her plan. HIV testing is a critical component of antenatal care in this setting, and in such high prevalence areas, regular testing, regardless of pregnancy status, should also be widely promoted. In Susan’s case, HIV testing prior to or earlier in her pregnancy could have led to more timely HIV treatment and a reduced risk for MTCT.

Detecting HIV is a first step to preventing further transmission. However, receiving a new diagnosis of HIV is a life altering event, and accepting the diagnosis, disclosing it to partners and family, and committing to lifelong treatment is emotionally and sometimes practically speaking, difficult. Denial and stigma often impede optimal adherence immediately after diagnosis. Indeed, several previous studies indicate that women newly diagnosed with HIV during pregnancy struggle with adherence and are less likely to remain engaged in care and sustain viral suppression [15, 23, 24]. Disclosing a new diagnosis of HIV can be extremely difficult, especially in cases where the partner’s status is unknown or HIV negative. Fears of stigma, being blamed, punished or being abandoned lead some women to withhold information about their HIV status from their partner and this directly impacts their medication adherence and engagement in care [13, 16, 25–27]. In Kenya, non-disclosure of HIV status to male partners has been associated with an increased risk for MTCT [28]. Unfortunately, despite frequent interactions with providers, Susan’s struggles to reveal her HIV diagnosis to her partner and adhere to ART were not recognized until the results of her viral load test came back at around nine weeks postpartum. During our first encounter with Susan, she reported perfect adherence to ART—despite later admitting to PMTCT clinic staff she had been throwing away her medications. Indeed, self-report can be an unreliable way to assess adherence [29]. Susan’s experience underscores the need for more intensive support for perinatal women newly diagnosed with HIV including support for disclosure to partners and more frequent objective monitoring of adherence/viral suppression through viral load testing. Other recent publications have also pointed to the need for increased viral load monitoring among pregnant and breastfeeding WLWH [30, 31].

This case of MTCT may have also occurred during labor and delivery. Kenya’s 2022 HIV prevention and treatment guidelines include ways to reduce the risk of HIV transmission during labor and birth such as minimizing vaginal examinations, using aseptic delivery techniques and avoiding artificial rupture of membranes [17]. Providers may consider these guidelines for WLWH, and HIV status is normally documented in the Mother and Child Health Handbook that mothers in Kenya typically bring to any hospital visit up to five years postpartum [32]. Yet, there is not always time for providers to review the handbook, and a lack of privacy in the maternity wards (where mothers often share beds) can leave women reluctant to discuss their HIV status with providers—this may have been the case for Susan. To complicate matters, Susan’s high viral load was yet to be discovered at the time of delivery, and neither infant PCR nor maternal viral load testing are routinely carried out at the time of birth in this setting [17]. Overall, it was not clear if any special considerations were made in managing Susan’s labor and delivery. However, improved communication with providers could have led to actions to reduce the chance of MTCT during labor and delivery.

Immediately after delivery, all women are encouraged to breastfeed their babies [18]. For WLWH, this should be after administering the first dose of HIV prophylaxis medication to their newborns [17]. Susan’s baby, who was seemingly healthy at birth, was exposed to HIV in breastmilk immediately postpartum without the protection of prophylaxis medications. In similar settings, infants who did not receive HIV prophylaxis medications were found to be at a higher risk of MTCT during breastfeeding [33, 34]. Thus, the failure to coordinate the provision of HIV prophylaxis medications to Susan’s son immediately after birth could have also been a factor in this case of MTCT.

Early testing and optimized treatment for infants exposed to HIV is part of the first pillar of the Global Alliance to end AIDS in Children. Indeed, the Lactation Specialist’s observations and communications with clinic providers which led to an early HIV test was an important factor for Susan’s son [4]. Yet, despite being tested at four weeks postpartum (compared to the routine testing at six weeks), it was not until Susan’s baby was about nine weeks old that she received the test results and her son was given ART. This type of delay in receiving and relaying test results is not uncommon. In fact, during this period, a shortage in laboratory reagents delayed infant PCR tests for most infants. HIV tests normally drawn at six weeks postpartum were not drawn until 8–10 weeks postpartum. Such a delay may have been detrimental to Susan’s son who could have benefited from testing and treatment even earlier. According to Kenya’s most recent HIV prevention and treatment guidelines (2022), infants at high risk of HIV acquisition (such as those born to WLWH whose viral load is unknown or who have been on ART for less than 12 weeks), should be considered for HIV testing immediately after birth. This guideline, which has yet to be fully implemented, would have been applicable in the case of Susan and her son. Moreover, the Lactation Specialist’s first referral to PMTCT clinic providers at just two weeks postpartum might have also prompted earlier testing and treatment given Susan’s diagnosis late in pregnancy and her baby’s symptoms. In addition to earlier HIV testing, using point of care testing with faster turnaround times has been shown to significantly reduce the time it takes to initiate ART for newly diagnosed infants [35].

HIV exposed infants have worse outcomes than infants born to women without the virus [36]. Therefore, optimal nutrition is of paramount importance for HIV exposed infants. However, women in this setting face many challenges adhering to infant feeding guidelines including food and financial insecurity, perceived breastmilk insufficiency and contrary cultural norms [18, 37–40]. Susan’s meetings with a Lactation Specialist early postpartum may have been an important factor not only through initiating early HIV testing but also by supporting Susan to sustain her exclusive breastfeeding practice. That Susan was able to maintain exclusive breastfeeding, was a protective factor for her son, as providing breastmilk only during the first six months is associated with reduced infant morbidity and mortality [41, 42].

This case sheds light on ways MTCT may still occur even when mothers are engaged in care. Yet, the rate of MTCT among women engaged in care is low, with one study in Kenya showing a transmission rate of just 2.5% among WLWH engaged in care at four Kenyan hospitals in 2016 [11]. Given most MTCT continues to occur among women *not* engaged in HIV care, more action is needed to understand and eliminate barriers to HIV testing and treatment for women and girls—particularly in sub-Saharan Africa where girls and young women 15–24 years old accounted for more than 77% of all new infections among this age group in 2022 [5, 7].

## Conclusions/ recommendations

We saw in this case report of MTCT that there were multiple points along the continuum of care that may have contributed to Susan’s infant contracting HIV. The perinatal period constitutes a major transition for all women and those living with and especially newly diagnosed with HIV are juggling additional challenges that require comprehensive, ongoing and personalized support. Early initiation/attendance of antenatal care clinic visits for timely HIV diagnosis and treatment, monitoring of adherence to ART through frequent and as needed viral load monitoring, support for disclosure to partners especially for discordant couples, coordination of perinatal and HIV care, early infant point of care testing and finally support for optimal infant feeding is necessary to strengthen efforts towards the elimination of MTCT and meet global commitments to end AIDS in children.

## Data Availability

Data on which this manuscript are based is not publicly available due to the need to protect privacy.

## Abbreviations

HIV: Human immunodeficiency Virus
MTCT: Mother-to-child transmission of HIV
PCR: Polymerase Chain Reaction
PMTCT: Prevention of mother-to-child transmission of HIV
UNICEF: United Nations Children’s Fund
WLWH: Women/ A Woman living with HIV
